# Regional protein expression in human Alzheimer’s brain correlates with disease severity

**DOI:** 10.1038/s42003-018-0254-9

**Published:** 2019-02-04

**Authors:** Jingshu Xu, Stefano Patassini, Nitin Rustogi, Isabel Riba-Garcia, Benjamin D. Hale, Alexander M Phillips, Henry Waldvogel, Robert Haines, Phil Bradbury, Adam Stevens, Richard L. M. Faull, Andrew W. Dowsey, Garth J. S. Cooper, Richard D. Unwin

**Affiliations:** 1 Division of Cardiovascular Sciences, School of Medical Sciences, Faculty of Biology, Medicine and Health, The University of Manchester, Manchester Academic Health Sciences Centre, Core Technology Facility (3rd Floor), 46 Grafton Street, Manchester, M13 9NT UK; 2 School of Biological Sciences, and Maurice Wilkins Centre for Molecular Biodiscovery, Faculty of Science, University of Auckland, Private Bag 92019, Auckland, 1142 New Zealand; 3 Department of Electrical Engineering and Electronics, University of Liverpool, Liverpool, L69 3GJ UK; 4 Centre for Brain Research, Faculty of Medical and Health Sciences, University of Auckland, Auckland, 1142 New Zealand; 5 Research IT, The University of Manchester, Manchester, M13 9PL UK; 6 Division of Developmental Biology & Medicine, School of Medical Sciences, Faculty of Biology, Medicine and Health, University of Manchester, Manchester Academic Health Sciences Centre, Manchester, M13 9PL UK; 7 Department of Population Health Sciences and Bristol Veterinary School, Faculty of Health Sciences, University of Bristol, Bristol, BS8 2BN UK

## Abstract

Alzheimer’s disease (AD) is a progressive neurodegenerative disorder that currently affects 36 million people worldwide with no effective treatment available. Development of AD follows a distinctive pattern in the brain and is poorly modelled in animals. Therefore, it is vital to widen the spatial scope of the study of AD and prioritise the study of human brains. Here we show that functionally distinct human brain regions display varying and region-specific changes in protein expression. These changes provide insights into the progression of disease, novel AD-related pathways, the presence of a gradient of protein expression change from less to more affected regions and a possibly protective protein expression profile in the cerebellum. This spatial proteomics analysis provides a framework which can underpin current research and open new avenues to enhance molecular understanding of AD pathophysiology, provide new targets for intervention and broaden the conceptual frameworks for future AD research.

## Abstract (web summary)

Jingshu Xu et al. did a comprehensive post-mortem proteomics analysis across six brain regions obtained from Alzheimer’s disease patients and controls. With this spatial proteomics approach, they identified new disease-related pathways and a gradient of protein expression changes that correlates with the pathology of the affected regions.

## Introduction

Alzheimer’s disease (AD) is a multifactorial neurodegenerative disorder characterised by progressive dementia^1,2^. Accumulation of Aβ peptide and microtubule-associated protein tau, which exhibits hyperphosphorylation, and oxidative modifications into so-called plaques and tangles are considered to be central to the pathology of AD^3^. Other prominent features of AD include early region-specific decline in glucose utilisation and mitochondrial dysfunction and consequently depleted ATP production and increased reactive oxygen species production in neurons^4^. Excitotoxicity in the AD brain arising from altered glutamatergic signalling^5^, and dysregulation in other neurotransmitters has also been documented, including abnormalities of adrenergic, serotonergic and dopaminergic neurotransmission^6^. In response to pathological stimuli associated with AD, inflammatory events mediated through both innate and cell-mediated immune mechanisms are also present^3^.

Despite an increase in research into the underlying pathology of AD over the last decade, there remains controversy around what underpins this disease process, which in turn affects the pipeline of new disease-modifying agents. There remains a lack of detailed mechanistic knowledge about what happens in the human brain in AD. This is exacerbated by the fact that different brain regions develop pathology at different times in the disease process, adding a spatial element to the disease, which is not captured by work in cell culture models and is often overlooked in human studies, which tend to focus on single regions. Animal models also fail to capture the full disease process, at either the behavioural or biochemical levels^7^, such that translation of both basic biological findings and/or the activity of potential disease-modifying interventions from animals into humans is relatively unsuccessful. While there have been several studies, which have focused on the transcriptome in human AD, there is a wealth of evidence that suggests many protein expression changes in biological systems can occur independently of transcript-level regulation, and that studying the proteome can provide new insights on the regulation of functionally active molecules in a given biological or disease state^8^.

Mass spectrometry-based proteomics has been recognised as a powerful tool with the potential to uncover detailed changes in protein expression^9^. To date, however, there are few studies of protein expression in AD carried out using human brain tissue, and those that exist typically examine a single AD-affected brain region^10,11^, and use different patient cohorts and analytical methods that makes between-region comparisons difficult. Such studies also frequently use either small numbers of samples (*n* < 4) or cohorts poorly matched for age or tissue post-mortem delay^10,12,13^. A recent study by Seyfried et al. bucks this trend somewhat by analysing larger numbers of brain samples from AD, asymptomatic AD (Braak IV) and control groups from two affected brain regions, the dorsolateral prefrontal cortex (FC) and precuneus (PC)^14^, and identifies functional networks present in these affected regions.

The current study aims to overcome some of these existing limitations by providing a spatially resolved analysis of protein expression in six regions of human control and AD-affected brain, reflecting varying levels of ‘affectedness’, in well-matched, short post-mortem delay tissue. Briefly, we quantify over 5000 proteins in AD and control tissue, to our knowledge the most in-depth study of this type to date. These data reveal protein changes between AD and control tissue, which appear to form a gradient through the brain, in order of affectedness where less affected regions display a smaller subset of those changes seen elsewhere, possibly representative of an early disease state. We also show that unaffected cerebellum, rather than being unaffected by AD, displays a pattern of protein expression changes distinct from other brain regions, which could be protective for this region of the brain.

## Results

### Study design

**Fig. 1:**
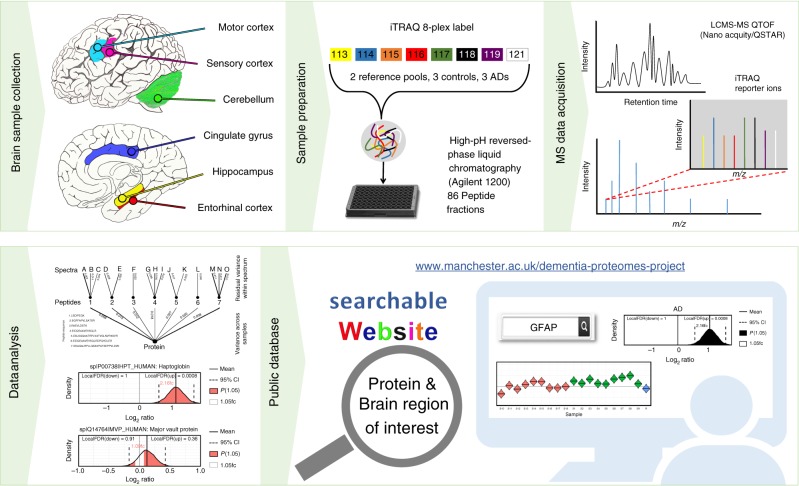
Proteomics workflow. Selected brain regions were pre-dissected prior to storage at –80 °C until analysis. Each region was lysed, and protein assigned to an iTRAQ 8-plex. Following digestion and labelling, samples were pooled, peptides fractionated by high-pH reverse phase chromatography and fractions analysed by standard LC-MS/MS methods. Peptides were identified and quantified based on their iTRAQ reporter area; relative protein quantification was inferred from these values using a Bayesian model. All data are deposited in a searchable online database

In this study, we analysed six functionally distinct regions of human post-mortem brain: hippocampus (HP), entorhinal cortex (ENT), cingulate gyrus (CG), sensory cortex (SCx), motor cortex (MCx) and cerebellum (CB), by mass spectrometry to gain a more comprehensive understanding of protein expression changes within the AD brain. These regions were selected to represent parts of the brain known to be heavily affected (HP, ENT, CG), lightly affected (SCx, MCx) and relatively ‘spared’ (CB) during the disease process. Donors (*n* = 9 AD cases, *n* = 9 asymptomatic controls) were well matched for age and post-mortem delay times were short, with no significant difference between cases and control. Donor data are provided in Table 1. Relative protein expression was determined using an isobaric tagging approach followed by two-dimensional liquid chromatography and mass spectrometry. Peptide-level data were then analysed using a Bayesian model that infers a posterior probability distribution for the relative levels of each protein between ‘cases’ and ‘controls’ based on the underlying relative peptide levels. To promote sharing and usage of these data, we have developed a searchable web interface that hosts all of our results (www.manchester.ac.uk/dementia-proteomes-project; described in Supplementary Information), which also includes Bayesian probability distributions for each protein across all individual brains examined in this study. The complete workflow is illustrated in Fig. 1. Raw mass spectral data can be accessed via PRIDE, with initial search outputs prior to Bayesian modelling available via the Open Science Framework at 10.17605/OSF.IO/6BXJQ (Supplementary Methods).

**Table 1:** Clinical characteristics of AD and control brains used in this study

Case no	Group	Age/sex	Ante-mortem brain/mental state	Cause of death	Braak stage	Amyloid load	PMD (h)	Brain weight (g)
1	AD	60/M	Alzheimer’s disease and dementia	Alzheimer’s disease	VI	3/3	7	1020
2	AD	62/F	Alzheimer’s disease and dementia	Alzheimer’s disease	VI	3/3	6	831
3	AD	63/F	Alzheimer’s disease and dementia	Bronchopneumonia	VI	2/3	7	1080
4	AD	70/F	Alzheimer’s disease and dementia	Lung cancer	V	3/3	7	1044
5	AD	73/M	Alzheimer’s disease and dementia	Gastrointestinal haemorrhage	IV	3/3	4	1287
6	AD	74/F	Alzheimer’s disease and dementia	Metastatic cancer	V	3/3	8.5	1062
7	AD	74/M	Alzheimer’s disease and dementia	Pseudomonas bacteraemia	VI	2/3	12	1355
8	AD	77/M	Alzheimer’s disease and dementia	Myocardial infarction	VI	3/3	4.5	1180
9	AD	80/M	Alzheimer’s disease and dementia	Bronchopneumonia/ pulmonary oedema	V	3/3	5.5	1039
10	Control	61/M	No brain disease or dementia	Ischaemic heart disease	-	0	7	1258
11	Control	64/F	No brain disease or dementia	Pulmonary embolism	-	0	5.5	1260
12	Control	63/F	No brain disease or dementia	Ruptured aorta	-	0	12	1280
13	Control	72/F	No brain disease or dementia	Emphysema	-	0	9	1230
14	Control	66/M	No brain disease or dementia	Ischaemic heart disease	-	0	9	1461
15	Control	76/F	No brain disease or dementia	Metastatic carcinoma	II	3/3^a^	12	1094
16	Control	73/M	No brain disease or dementia	Ischaemic heart disease	-	0	13	1315
17	Control	78/M	No brain disease or dementia	Ruptured abdominal aortic aneurysm	-	0	7.5	1260
18	Control	78/M	No brain disease or dementia	Ruptured myocardial infarction	-	0	12	1416

Brain pathology and amyloid load, were determined using the scoring system based on Braak and Braak staging, where a score out of 3 was determined by a qualified neuropathologist and cause of death was determined at post-mortem examination. ^a^Despite being phenotypically healthy, patient 15 was found retrospectively to have post-mortem signs consistent with AD and was described as A3, B1, C1 using the ‘ABC’ criteria for AD neuropathologic change that incorporates histopathological assessments of Aβ deposits (A), staging of neurofibrillary tangles (B) and scoring of neuritic plaques (C). The corresponding data have been retained in the analysis presented in the article due to the early and asymptomatic nature of this patient

AD: Alzheimer’s disease, F: female, M: male

### Regional comparison of protein expression in human AD brain

Each brain region was analysed in isolation, adding strength to our comparison of protein expression changes across multiple regions, since these were identified and quantified independently. An initial principal components analysis (PCA) of the data for each region shows that samples appear to separate on the basis of disease class (Supplementary Figure 1a)—there is no significant clustering and hence confounding by age, sex or post-mortem delay (PMD) in this analysis overall. In addition, we did not observe any significant gender clustering in the AD cases when analysed in isolation (Supplementary Figure 1b), as may be anticipated given the well-matched nature of the case and control sample sets.

**Fig. 2:**
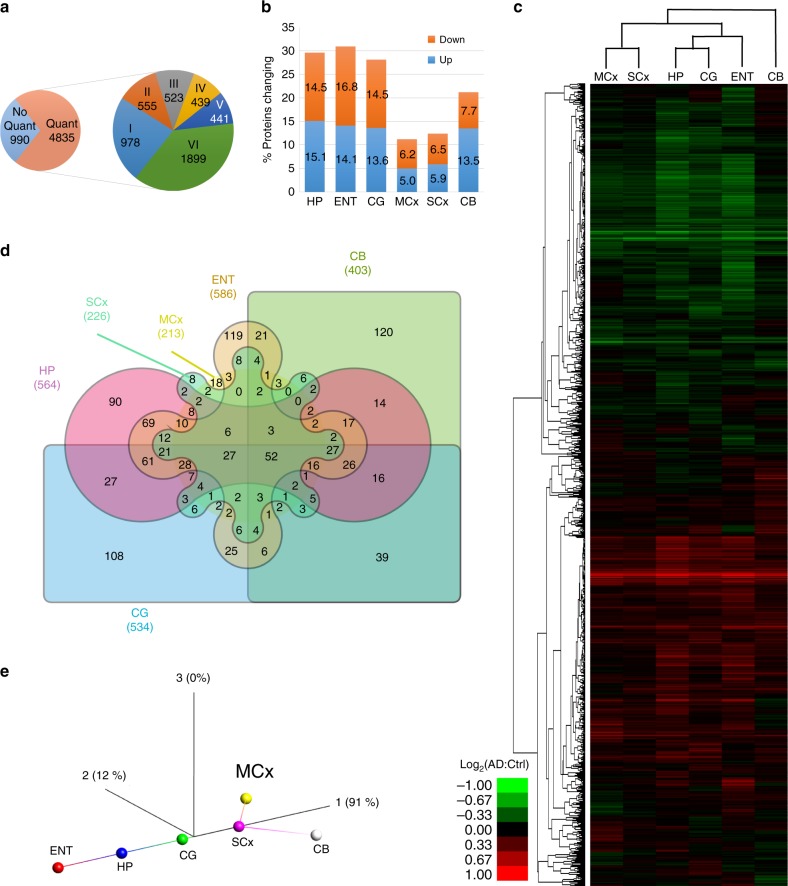
Summary of protein expression data. **a** In total, 5825 proteins were identified, with 990 quantified with only one or two spectra and which were thus omitted from our primary comparative analysis. The remaining 4835 proteins are classified as to whether they were quantified in six or fewer distinct regions. **b** Proportion of identified, quantified proteins showing a change in expression in Alzheimer’s disease (AD) in each of the six regions under study. **c** Heat map and dendrogram showing the relationship between protein expression in each region mapped using proteins present in all six regions, with three distinct ‘groups’ based on highly affected (hippocampus (HP), entorhinal cortex (ENT), cingulate gyrus (CG)), moderate (motor cortex (MCx), sensory cortex (SCx)) and spared (cerebellum (CB)) clearly visible. **d** Edwards–Venn diagram showing the overlap of protein expression changes between brain regions, including only proteins quantified in all regions. **e** Isometric mapping (Isomap) representation of protein expression data between brain regions showing correlation in protein expression from non-affected towards affected regions, with the exception of cerebellum, which shows distinct patterns of protein expression in AD

Combining all protein identifications (at 1% false discovery rate (FDR)) across the six experiments yielded a total of 5825 unique protein identifications across all regions. The complete processed data for each region (at protein identification FDR < 1%) can be found in Supplementary Data 1. In our data, 990 proteins were quantified with only one or two spectra in any single region, and were subsequently omitted from our downstream cross-regional comparison in order to retain the proteins with the most precise quantification—optimisation data suggest that when the same sample is split and processed independently, > 99% of proteins are defined as not being significantly different above this threshold (A. W. Dowsey, personal communication). However, many of these will be quantified correctly (we have previously validated expression changes based on a single spectrum, e.g., p53 in^8^), and as such these data have been included in Supplementary Data 1 and our online database. We thus quantified a total of 4835 distinct proteins in at least one brain region, among which 3302 proteins were common to at least three regions, and 1899 to all six regions (Fig. 2a). These data allow us to (a) define protein changes as a result of AD in any given region of the human brain being studied, and (b) identify differences in how distinct brain regions are affected in AD, and by extension protein changes, which occur in multiple regions of the AD brain.

Comparison of the total number of proteins whose expression is altered in each region reveals, perhaps unsurprisingly, that the more severely affected areas in AD (HP, ENT, CG) show the largest number of changes in protein expression (~30% of quantified proteins), whereas less affected regions (MCx, SCx) have fewer changes (11–13%). Strikingly, the CB, which many think to be pathologically ‘unaffected’, shows a substantial number of protein changes (20%; Fig. 2b). This observation accurately recapitulates data from our previous study of the metabolome on these brain samples^15^. Unsupervised hierarchical clustering of protein expression changes from all six regions demonstrates that the changes observed in CB are distinct from those seen in the affected HP, CG and ENT (Fig. 2c). This is supported by an Edwards–Venn representation of the data, which shows that 120/403 (29.8%) of changes in CB are not seen elsewhere (Fig. 2d; Supplementary Data 2). While it has long been reported that the CB in AD can contain amyloid plaques^16^, it is considered to be relatively ‘spared’ in AD. There is a lack of neurofibrillary tangles in CB^17^, and this region does not appear to develop notable neuronal loss, such that this region is often used as a control in imaging studies of the AD brain^18,19^. However, recent work by Guo et al. suggests a distinct pattern of cerebellar atrophy, which spreads from intrinsic connectivity networks within the cerebrum^20^, and alterations in cerebellar glucose metabolism have been reported in late stages of the disease^21,22^. Our data strongly suggest that the CB is heavily affected by AD at the molecular level, at least in late stage disease, and is so to a greater extent than other regions associated with later degeneration such as MCx or SCx, where protein changes were fewer and encompass those seen in the more severely affected regions. That the changes in CB are different from those seen elsewhere in the brain raises the possibility that, rather than being ‘spared’, the CB is affected in a different way to other brain regions and that, given it shows little pathology, these changes may reflect some level of active protection.

Hereinafter, we refer to HP, ENT and CG as the severely affected, and MCx and SCx as the less affected regions based on the number of significantly altered proteins and pathways observed within this study.

Unsupervised clustering of brain regions based on their protein expression, by performing a dimensionality reduction on these data using isomeric feature mapping (Isomap), clearly shows this hypothesised ‘evolution’ of the disease from the least affected cortical regions to the most affected, with CB following a distinct pathway from the inception of disease (Fig. 2e). This non-linear approach has been shown to be an improvement over the more standard PCA approach for analysis of gene and signalling networks^23^. These data also further support our previous observation that CB stands out as a single, uniquely affected brain region based on the distinctive patterns of changes found here, whereas the other regions line up along the same vector in accordance with disease severity.

Previous studies using gene co-expression networks and transcriptomics analysis have demonstrated a pattern where the molecular signatures in less affected areas of the brain overlap with but are less marked than the grossly affected areas, and have hypothesised that these regions are on a different point along a continuum of disease progression^24^. As such changes in less affected areas (which are mirrored in the highly affected areas) likely represent those which occur early in AD-related neurodegeneration^24^. Our data at the protein level would support this conclusion—the less affected regions (MCx and SCx) contain very few protein changes, which are not seen elsewhere. An unsupervised clustering analysis suggests that these regions are simply at an earlier stage down a similar pathway. This supports the hypothesis proposed by Ray and Zhang that by comparing more and less affected brain regions in a multi-regional approach we can observe different stages of the this progressive disease, enabling identification of early molecular changes.

### Pathways dysregulated in human AD

To identify key protein expression changes in the brain in AD, we first identified all proteins, which show differential expression in at least 5/6 brain regions. This subset was selected as these proteins are guaranteed to be changed in at least one of MCx and SCx, and as such likely also represent changes, which occur earlier in disease, and are thus more interesting from a therapeutic targeting perspective. The 128 proteins, which fit this criterion are listed in Supplementary Data 3. We can find no prior evidence in the literature that 44 of these proteins have been previously linked to AD. These are novel findings and include proteins involved in the protein folding/stress response, in metabolism, in neurotransmitter production and exocytosis, and in cell signalling. A further 22 of these proteins have only been previously linked to AD via other -omics studies, including another recent large-scale human brain proteome analysis^14^ and several others have only been linked to AD via studies on animal models, and so our dataset provides valuable validation data for these proteins in human disease tissue.

**Fig. 3:**
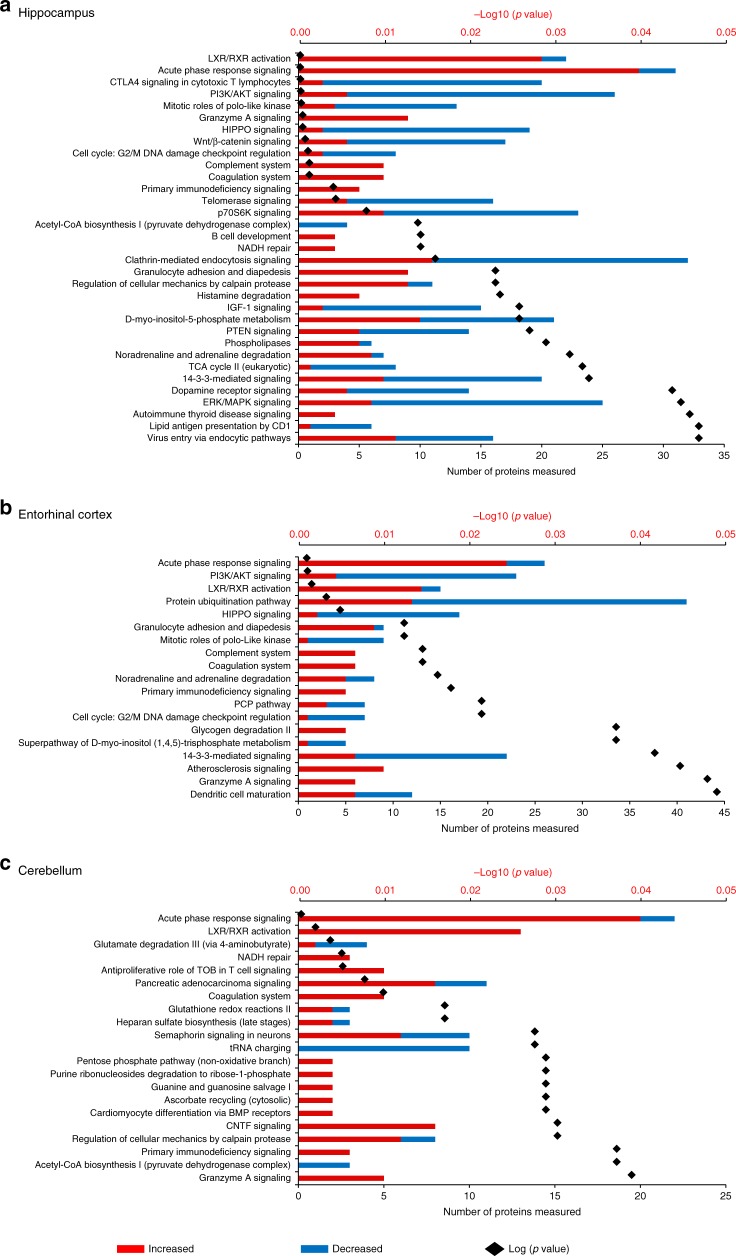
Network analysis summary. Alterations of molecular pathways in human Alzheimer’s disease brain across six distinct regions, namely **a** hippocampus, **b** entorhinal cortex and **c** cerebellum. In each plot, the numbers of increased and decreased proteins are indicated by the red/blue bars, whereas the black spots indicate the log10 (*p*-value) for each pathway

To probe the differences in AD-related protein expression between brain regions in more mechanistic detail, we performed a pathway enrichment analysis for all differentially expressed proteins for each region. Such analyses enable us to visualise which processes are affected in the AD brain, and also whether two (or more) regions are showing dysregulation in the same pathway even if different subsets of proteins are identified as ‘changing’. These data are summarised in Fig. 3a and Fig. 3b (and Supplementary Data 4).

Reflecting the individual protein expression data, HP and CG showed the highest number of biological pathways being affected by AD. The changes in specific molecular pathways were comparable between HP, ENT and CG. CB, on the other hand, showed altered regulation of a set of molecular pathways with limited overlap with those affected in the other five brain regions, again arguing for the presence of a distinct cellular response to disease in this region.

**Fig. 4:**
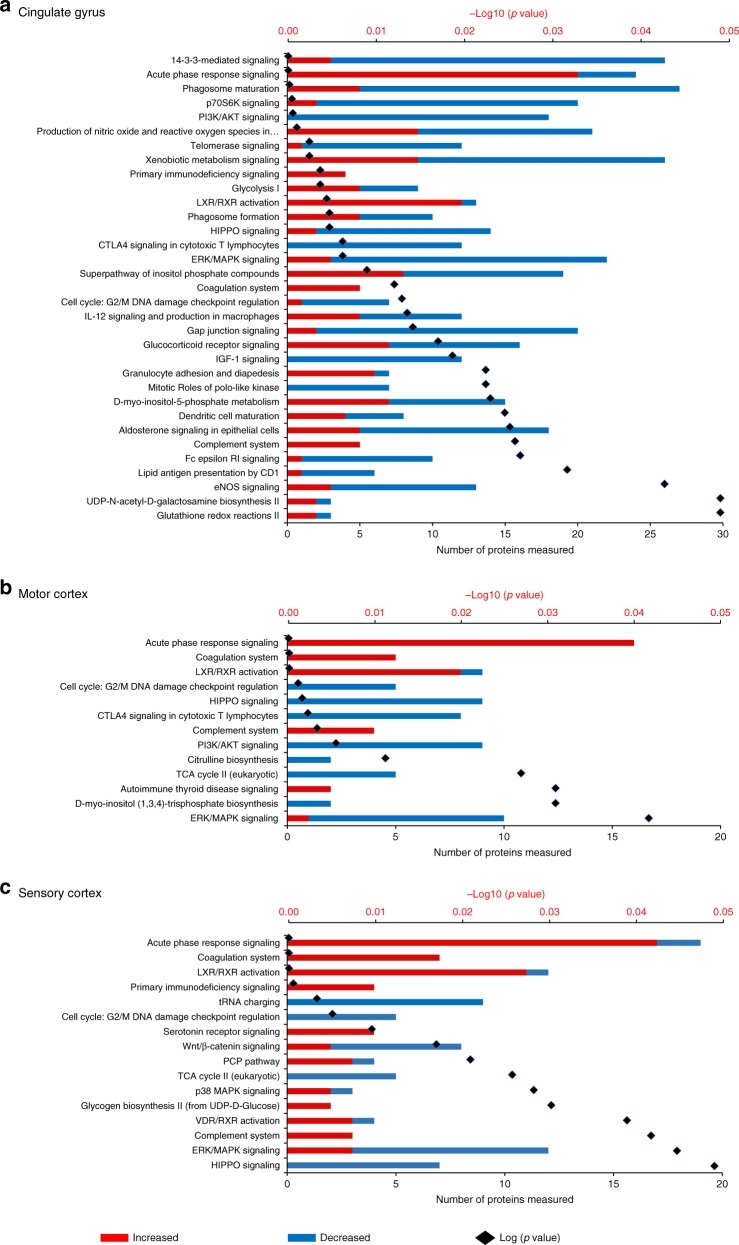
Network analysis summary. Alterations of molecular pathways in human Alzheimer’s disease brain across the: **a** Cingulate gyrus, **b** Motor cortex, and **c** Sensory cortex. In each plot, the numbers of increased and decreased proteins are indicated by the red/blue bars, while the black spots indicate the log10 (*p*-value) for each pathway

One of the most consistent features across all brain regions was a significant change in proteins and pathways involved with the innate immune response. In AD, aggregates of Aβ can trigger both pathogen-associated and initiate immune responses, and a persisting elevation of Aβ may elicit a chronic reaction of the innate immune system^25^. In this study, we observed strong evidence for the global activation of the innate immune response, including of the acute phase response, the complement system (classical and alternative pathways) and the coagulation system, consistent with widespread neuroinflammation, suggesting that this may be a relatively early (prior to atrophy) event in pathogenesis. Previous studies have also implicated complement family proteins as potential AD biomarkers^26^, and genome-wide association studies have identified AD risk loci in a number of complement pathway genes^27–29^. It is worthy of note that these studies do not directly inform on the activation state of the complement pathway, and indeed in our study we see upgregulation of SerpinG1, which inhibits complement C4 cleavage by C1 and MASP2, as well as increased levels of C4, C3 and various regulators in AD. Although it is highly likely that dysregulation of this pathway plays a role in AD, the precise nature of this role remains to be determined. Overall, HP, ENT and CG showed substantive evidence for a broader spectrum of changes in immune responses compared with MCx, SCx and CB. These included specific cellular pathways including granulocyte adhesion and dendritic cell maturation (Figs. 3 and 4, Supplementary Datas 3 and 4), implying that while the innate immune system becomes activated throughout the brain, the adaptive immune response is primarily activated in regions of more significant damage. This supports our previous hypothesis, and that of Ray and Zhang^24^ who noted a similar disparity in immune processes between less and more affected regions that these regions lie on a continuum of disease, and that what we are observing is that while regions, which are earlier along this continuum have activation of innate immunity, adaptive processes are only present late in disease, possible as a response to cellular damage. However, the interplay between these two systems is complex and it is yet to be determined if these changes are a cause, or a consequence of other aspects of AD pathogenesis^30^_._

This pathway-level analysis also identified signalling pathways involved in apoptosis and cell cycle regulation as being widely dysregulated in severely affected regions of AD brain, including the HIPPO, ERK/MAPK, PI3K/AKT and Wnt/β-catenin pathways (Figs. 3 and 4), all known to be critically involved in regulation of apoptosis and the cell cycle. Reduced abundance of proteins involved in Polo-like kinase signalling and G2/M DNA damage checkpoint regulation are likely a cause of impaired cell cycle regulation, marking these pathways out as potentially key contributors to neuronal cell death in AD. Strikingly, less affected regions SCx and MCx do not show large changes in these pathways, reflecting reduced levels of apoptosis seen in these areas and providing further support for the idea that these regions are reflecting ‘early’ disease changes. In CB, only granzyme A signalling was identified as an apoptosis-related pathway, indicative of fewer cell death signals in this region.

The only exceptions are the G2/M checkpoint and the Hippo pathway, whose members are significantly decreased in these regions, suggesting that inactivation of this key developmental pathway, possibly via the observed upregulation of CD44^31^, or altered regulation of associated proteins such as the synaptic scaffolding proteins DLG2, DLG3 and DLG4, all of which are downregulated, is an early event in AD development. The observation of an altered Hippo signalling pathway in all areas of the brain studied is, to our knowledge, the first time that this pathway has been directly implicated in AD, although it has previously been shown that the human orthologue of Hippo, MST1, phosphorylates Foxo3 and that this is required for neuronal death due to presence of reactive oxygen species (ROS) or lack of neurotrophic activity^32^. This pathway can be also activated by amyloid beta in primary cortical neuron cultures^33^. The Hippo pathway is thought to be primarily involved in the regulation of organ size and developmental processes within the brain. However, links to neurodegeneration in ALS^34^, and a role in microglial activation following ischaemic stroke^35^ suggest that it is worthy of more investigation into any potential role in the early stages of AD.

**Fig. 5:**
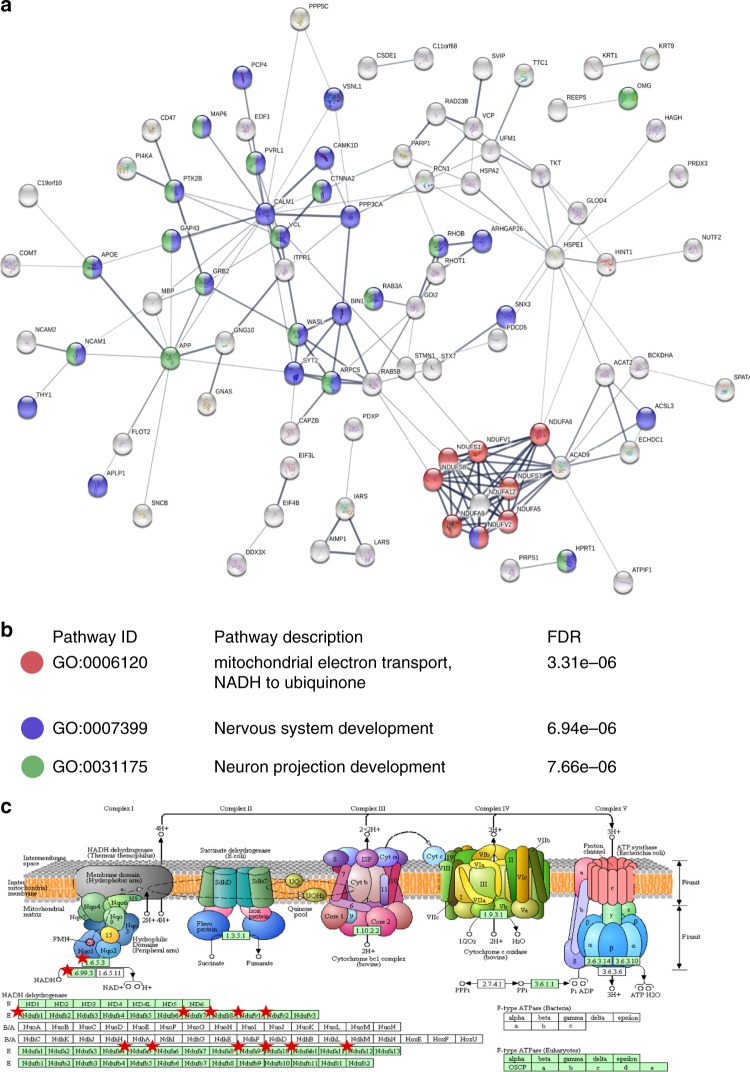
Cerebellum (CB)-specific biological processes in Alzheimer’s disease (AD) brain. **a** In total, 120 proteins that showed CB-specific alterations were enriched for molecular processes in STRING using default setting. Each node represents a protein, and proteins involved in **b** significantly enriched pathways were highlighted. **c** Dysregulation of the mitochondrial electron transport chain was highlighted by pathway analysis, and proteins affected mapped (red star) into the NADH dehydrogenase complex in KEGG oxidative phosphorylation map

We also observed both global and regional metabolic impairments in the AD brain. Defects in brain metabolism and energetics are central to the pathogenesis of AD as evidence by epidemiological, neuropathological and functional neuroimaging studies^36^. The AD brain characteristically exhibits defective cerebral perfusion^37^ and glucose uptake^38^, which is believed to underlie hypometabolism and cognitive decline^39^. Alterations in pathways of monosaccharide/glucose metabolism are highly significant in severely affected brain regions and CB (Fig. 5, Supplementary Data 3), consistent with our previous finding of elevated free glucose levels in AD brain^22^. Citric acid cycle  enzyme abundance was generally decreased in all regions of AD brain, going some way to explaining the previously observed shift from primarily aerobic glycolysis (i.e., glycolysis followed by complete oxidation in mitochondria) to the ketogenic/fatty acid β-oxidation pathway, with impaired mitochondrial bioenergetics^40^. Severely affected brain regions also showed substantial alterations in signals related to altered regulation of neurotransmitters/hormones (noradrenaline/adrenaline, dopamine and aldosterone) that were not observed in less affected regions. Although this might suggest that altered neurotransmitter biology is a late or downstream process in pathogenesis, it is notable that enzymes in the Tetrahydrobiopterin (BH4) pathway, a key upstream pathway of neurotransmitter production are differentially expressed in all regions. BH4 acts as a substrate for the production of several neurotransmitters, including dopamine and serotonin. Three proteins, SPR, QDPR and PCDB1, which catalyse the conversion of BH4 away from these neurotransmitters and towards biopterin increase throughout the brain. This is the first time that these proteins have been observed to be defective in AD brain, although reports from the mid-1980s demonstrated reduced BH4 in AD^41^. This is the first time that enzymes from this pathway have been directly implicated in AD pathogenesis, although previous work has suggested a decrease in BH4 levels in AD brain^42^. The observations at the protein level may reflect either a feedback loop where the cell is responding to decreased BH4, or a shift in BH4 metabolism towards biopterin and away from NT production. The presence of this dysregulation early in disease suggests it is a target, which deserves closer attention.

The pathways we have identified as changing in AD share some overlap with those identified in a recent study by Seyfried et al.^14^. Here two brain regions, dorsolateral FC and PC were compared for patients from AD, asymptomatic AD and control groups. Taking the data from their paper and processing it using the same pathway analysis tools as used to analyse our data yields some interesting observations. Despite the presence of a small number of protein changes (63) between the asymptomatic and control groups (at *p* < 0.01, as used in the original publication), no significant pathways could be determined for this subset, despite the presence of Braak IV pathology in most of these samples. Analysis of the Seyfried AD vs control data from FC identified pathways also seen in our data, including a range of overlapping signalling pathways around actin cytoskeleton signalling and cell motility, synaptic long-term potentiation, semaphorin signalling and myoinositol metabolism (Supplementary Data 5A). Fewer pathways were seen in the dataset from PC. Notable by their absence, however, were the strong signals, which we observed from neuroinflammatory pathways and metabolism, although ‘inflammation’ was a feature of one of the protein co-expression modules extracted from the Seyfried data. A search of these data suggests that most of the proteins, which we found to be differentially expressed in the ‘acute phase response’ signalling pathway were quantified by Seyfried et al., but did not show differential expression. Similarly of the eight proteins from the citric acid cycle, which we showed to be differentially expressed (IDH3A, IDH3B, OGDH, OGDHL, IDH3G, ACO1, SUCLA2, SUCLG1), all were identified by Seyfried but none differed significantly between AD and control. This is surprising given that these changes are well established in human AD. The reasons for these disparities are unknown, although of course the two studies are investigating different regions of the AD brain. Of the 250 proteins identified as being differentially expressed in FC by Seyfried at al., which were also identified in our study, 162 (65%) were differentially expressed in both studies (Supplementary Data 5B).

Although comparison of affected regions yields a range of interesting and novel observations about the molecular underpinning of AD, the presence of a large number of changes in ‘unaffected’ CB provides a surprising finding, even more so when one observes that these changes are distinct from those manifest elsewhere. To investigate this population of protein changes further, we analysed proteins uniquely affected in CB using both DAVID and STRING. These analyses supported our earlier global pathway analysis in demonstrating that CB additionally showed alteration in Semaphorin and ciliary neurotrophic factor pathway members, which play important roles in neuronal survival and neurodevelopment/neuronal regeneration (Figs. 3 and 4). SEMA7A, shown here to be upregulated in CB of AD brains, is known to be involved in repair of the glial scar following spinal cord injury and to play a role in the development of multiple sclerosis, but has not previously been linked to the disease process in AD^43^. CB also showed a significant reduction in levels of both nuclear and mitochondrial aminoacyl tRNA (transfer RNA) synthetases. In CB, significantly depleted aminoacyl tRNA synthetases, including those encoded in the mitochondrial genome, as well as those from the nuclear genome (Figs. 3, and 4 and Supplementary Data 2), could disrupt translational fidelity, leading to accumulation of misfolded proteins^44^. However, these proteins are multifunctional. For example, Ishimura et al. have shown that dysregulated tRNA processing can lead to neurodegeneration^45^, and tRNA synthetases have also been shown to be mediators of inflammation^46^ thus downregulating these proteins may confer some level of protection. This finding could also provide a supportive mechanism for the hypothesis that ribosomal dysfunction is an early event in AD^47^. Taken together with its known roles in inflammation and signalling, and in several other neurodegenerative disorders^48^, our data suggest that the role of tRNA synthetases in AD is worthy of significant further investigation.

One of the most distinct changes observed in this CB-specific analysis was that a much greater number of proteins of electron transport chain (ETC) complex 1 were consistently more reduced in abundance (Fig. 5; Supplementary Data 6) than was found in other areas. Furthermore, CB showed increases in oxidative defence proteins involved in glutathione redox reactions and ascorbate recycling (Figs. 3 and 4). These data provide strong additional evidence for a protective mechanism in CB that decreases ROS production by ETC while simultaneously increasing ROS defences. Another interesting observation in CB was the activation of a purine ribonucleosides degradation pathway, which could not only contribute substrate to the pentose phosphate pathway, but also participate in guanine/guanosine production in this brain region. Combined with the observed activation of Guanine and Guanosine Salvage I pathway, and an increase in guanosine level in CB as previously reported by our metabolomics analysis^15^, these changes may also confer a previously unknown neuroprotective effect in this brain region^49^.

It is well established that CB does not display extensive apoptotic activation seen elsewhere in the brain in AD, which is unsurprising given its structurally unaffected status. Our findings indicate that the lack of significant neurodegeneration in this region is not merely due to the absence of an apoptotic signal (e.g., Tau tangles) but instead that CB actively induces a unique pattern of upregulated neuronal survival pathways alongside protection against oxidative and inflammatory damage; a protective mechanism of gene/protein expression, which limits disease-related degeneration in this region.

### Key regulators of AD-induced protein expression changes

**Fig. 6:**
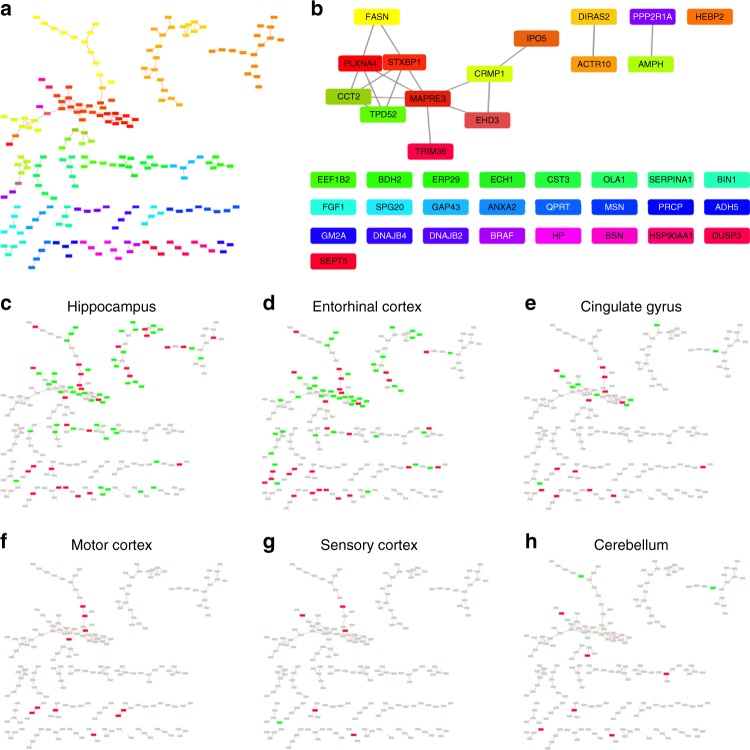
Global networks analysis was performed using Cytoscape ModuLand plug-in. **a** Correlation network of altered proteins in Alzheimer’s disease (AD) brain, with differently coloured clusters representing different meta-nodes. **b** Key regulators of each meta-node based on intra-network connectivity. **c**–**h** Overlays of protein expression data from each region and the correlation network

Given the apparently similarity in protein expression, which we seen within each group (severely affected and less affected), we next attempt to identify key regulators of what appears to be a coordinated alteration in protein expression across the brain in response to AD. We performed a correlation network analysis to identify key nodes, which may be responsible for the programme of protein expression observed, using the Cytoscape ModuLand plug-in^50^. The resulting correlation network is shown in Fig. 6a. Each cluster is coloured differently according to a distinct meta-node, the key regulators of which can be determined by visualising higher levels of this hierarchy (Fig. 6b). Using this method, we can identify the most influential genes in this correlation network, which we hypothesise to be key regulators of protein expression during the pathogenesis of AD. It is noteworthy that in this correlation matrix we are aiming to correlate what we believe to be two distinct processes—AD pathogenesis (seen in HP, ENT, CG, MCx and SCx) and a protective programme that we observe in CB. By overlaying protein expression data onto this network, we can identify which nodes are associated with which process. This overlay (Figs. 6c–h) clearly demonstrates that the correlation network is mainly constructed from proteins involved in AD pathogenesis in the affected regions—few proteins in the network are changed in CB despite the relatively large number of CB proteins, which we observe to be changed in the complete dataset. This is to be expected as CB-specific protein changes have limited correlation to the remainder of the dataset. This network is therefore likely to provide a good representation of the key events in AD pathogenesis, and reveals four proteins with the most overall influence on the correlated expression networks, based on intra-network connectivity: syntaxin binding protein 1 (STXBP1); collapsin response-mediator protein 1 (CRMP1); actin-related protein 10 homologue (ACTR10); and amphiphysin (AMPH).

STXBP1 is the regulator with the most influence in this network. It is reportedly upregulated in AD^51^, has been linked to NFTs^52^ and may interact with PS1^53^. It also plays a major role in neurotransmitter release. STXBP1 thus provides a potential mechanistic explanation for our observation that pathways of neurotransmitter metabolism including dopamine-, noradrenaline- and serotonin-related signalling showed significant changes in severely affected regions and SCx, but not in MCx or CB. Another important regulator of the network, CRMP1, is part of the semaphorin signalling pathway, which is known to guide axons in developing nervous tissue and participates in shaping of neural circuits^54^. ACTR10 may affect prion susceptibility through its involvement in prion propagation and clearance^55^, and has been identified by large-scale computational network analyses as one of a large number of potentially important genes in hippocampal ageing, but our finding is novel in AD^56^. The fourth key network regulator identified here, AMPH, is a candidate AD risk gene that may participate in receptor-mediated endocytosis and hence be involved in APP metabolism/clearance^57^. Our finding that these four genes appear to be central to various pathological processes known to be involved in AD development is important, and suggests that further work should be performed to focus on the role of these potentially key mediators of AD progression.

### Measurement of amyloid beta

**Fig. 7:**
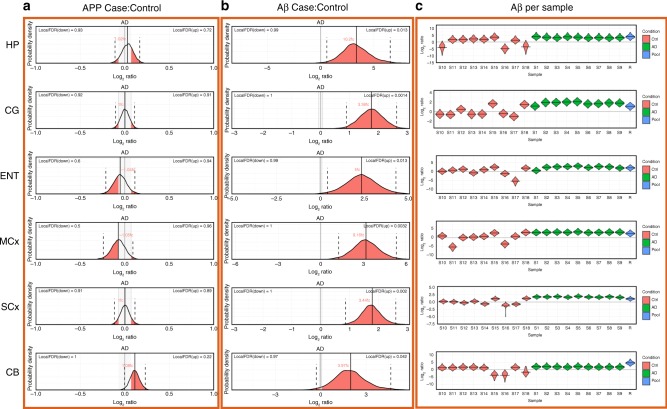
Expression of amyloid precursor protein and Aβ peptide. Expression probability distributions for **a** amyloid precursor protein and **b** the Aβ peptide (sum of 1–40 and 1–42 peptides, which are not distinguishable in this assay) for all brain regions. Each plot shows the probability distribution along with the most likely mean expression ratio and the calculated false discovery rate (FDR; 1-probability) for each molecule having a differential expression between cases and control of at least 5%. **c** Bayesian probability distributions for estimated levels of amyloid beta peptides 1–40 plus 1–42 ion each individual sample in this study

As one of the key factors in AD pathogenesis is thought to be the build-up of amyloid consisting of Aβ peptide generated as a proteolytic product of the amyloid precursor protein (APP) we examined our data for information about the levels and distribution of these molecules. We found no marked change in APP levels overall but significantly elevated Aβ peptide levels (Fig. 7a, b), consistent with previous reports^14^. The extent of the increase in Aβ between regions does not appear to follow a gradient of ‘affectedness’, albeit there may be a more pronounced increase in HP. Indeed Aβ levels are increased in CB, despite the differential response observed in this region. There is no way to determine the primary structure of the Aβ peptide(s) present in each region from these data. Interestingly, whereas in the AD group almost all samples showed uniformly high levels of Aβ peptide, there was marked variation in levels in control samples (Fig. 7c). Although the quantification of Aβ is necessarily from one peptide, these data emanate from between 5 and 12 unique spectra in each sample, we consider this observation is likely robust. This variability is therefore likely to be due to inherent variations in the control population.

Although all patients in this group were asymptomatic, it is likely that varying degrees of prodromal disease could have been present, given their age. This is most noticeable in our control #15. While initially assigned as a control, a pathological re-examination performed as a result of the findings of this study and our previous metabolomics analyses^15^ re-classified this individual as a Braak II preclinical AD patient. This patient has the highest level of Aβ of all of the control samples and interestingly appears to demonstrate some AD-related changes both in their metabolome and in some of the proteins, which we observe to be changed in symptomatic disease, although drawing any conclusions from this single case would be ill-advised at this stage. However, this sample was retained in the analysis, both for our previous metabolomics^15^ and metallomics^58^ studies on this cohort and for the current study, since the donor was asymptomatic at the time of death, and therefore remains representative of a ‘non-AD’ population in this age cohort. This decision is supported by PCA analysis of both metabolomics and proteomics data (Supplementary Figure 1), which suggests that this sample clusters more closely with the control samples than AD in most regions. This observation supports the idea that increases in Aβ levels may reflect varying degrees of prodromal disease in these elderly controls. It also demonstrates that studies of the type performed here in earlier stage presymptomatic patients will be critical to further tease out the very earliest events in AD pathogenesis.

## Discussion

In summary, this study provides a map of molecular changes that are present in human post-mortem brain tissue in patients with AD and matched controls, providing insights into the brain region specificity of disease at two levels; individual proteins and pathways. We observed global perturbation of protein expression in all six regions of the AD brain that we studied. An association between extent of molecular changes and affectedness was observed for five regions, allowing us to delineate probably ‘early’ and ‘late’ changes in protein expression and revealing previously novel involvement of several pathways and processes. The sixth region, CB, showed an unexpectedly distinct pattern of protein changes, suggestive of induction of a protective response. Correlation network analysis identified four candidate genes STXBP1, CRMP1, ACTR10 and AMPH, which may underpin significant portions of the protein expression response to AD. Finally, we recognise that these data have significant value to the community and that other researchers will no doubt wish to assess the status of other AD-related changes not discussed here. As such we have provided all results in an accessible format via a freely available, searchable online database, to allow others to probe specific pathways or individual proteins and their expression in regions across the human AD brain and matched controls.

## Methods

### Human brains

All experiments were performed in accordance with relevant guidelines and regulations. The case–control study of post-mortem human brain was approved by the University of Auckland Human Participants Ethics Committee with informed consent from all families.

Human brains were obtained from the New Zealand Neurological Foundation Human Brain Bank, University of Auckland^59^. Each brain was dissected under the supervision of neuroanatomists (J.X., S.P., H.W. and R.L.M.F.), who accurately identified each region as previously described^22^. Brain regions studied were HP, ENT, CG, SCx, MCx and CB: grey matter from each region was sampled. Aliquots of 100 ± 5 mg were dissected from each region and stored at –80 ^o^C until analysis, and were otherwise treated as previously described^60^. Patients had ante-mortem evidence of clinical dementia, whereas controls did not. Controls were selected by matching for age, sex and post-mortem delay. A consultant neuropathologist diagnosed or excluded AD by applying the Consortium to Establish a Registry for Alzheimer’s Disease (CERAD) criteria^61^, and determined the neuropathological severity by assigning the Braak stage^62^ and amyloid load by applying the 2013 consensus National Institute on Aging–Alzheimer’s Association guidelines^63^ (Supplementary Data 1). One control patient (115) had neuropathological findings consistent with AD (Braak Stage II; Supplementary Data 1) and was therefore diagnosed with preclinical disease: this finding is consistent with the known frequency of asymptomatic AD in similarly aged groups in the study population^64^.

### Protein extraction and preparation for iTRAQ labelling

Protein extraction and preparation for iTRAQ was carried out according to a previously described method^65^, with each brain region analysed independently. Brain tissue samples of 100 ± 5 mg were extracted in 500 μL 1 M Triethylammonium bicarbonate buffer (TEAB) + 0.1% (w/v) sodium dodecyl sulphate (SDS), and homogenised at 25 Hz (2 × 3 min) with a Qiagen tissuelyser. The tubes were then vortexed for 10 s and centrifuged at 4 ^°^C for 5 min at 13,400 × *g*. The supernatants were transferred into a new set of tubes and protein concentration was determined by using Bradford protein assay (Bio-Rad Protein Assay Dye Reagent Concentrate) and a SpectraMax M5 plate-reader (Molecular Devices). From each sample, a volume equivalent to 100 μg protein was transferred into a new set of tubes for further processing. Identical reference pool samples (total of 100 μg protein per reference sample) were made by combining portions from four representative individual samples from each group, AD and control. All samples were equalised for final volume using 1 M TEAB + 0.1% (w/v) SDS.

Protein samples were reduced by addition of 0.1 volume of 50 mM dithiothreitol, followed by incubation at 60 °C for 30 min. Alkylation was carried out by addition of 0.05 volumes of 200 mM iodoacetamide, followed by incubation in the dark at room temperature for 10–15 min. Protein digestion was subsequently carried out overnight at 37 °C, by adding 10 μg of modified porcine trypsin (Promega) re-suspended in 1 M TEAB, ensuring the final SDS concentration fell below 0.05% (w/v). After digestion, the samples were dried completely in an Eppendorf concentrator, and re-suspended in 30 μL 1 M TEAB to achieve equal volume across all samples before iTRAQ labelling. The iTRAQ labelling was carried out according to the manufacturer’s instruction using the 8-plex iTRAQ kit (AB Sciex). Briefly, vials containing iTRAQ reagent were thawed on the bench for 2–3 min. After spinning the samples down, 70 µL isopropanol was added to each vial, followed by a pulse spin. The content of the vials was then transferred to the protein samples and then incubated on the bench for 2–3 h. Each 8-plex contained two separate digests of the reference pool sample, three AD samples and three control samples. iTRAQ-labelled samples destined for the same liquid chromatography/tandem mass spectrometry (LC-MS/MS) run were pooled, followed by a spin at 13,400 × *g* for 5 min. Each pooled sample was then divided into two equal aliquots and dried completely using an Eppendorf centrifugal evaporator concentrator. One pooled aliquot from each 8-plex experiment was subjected to high-pH reverse phase (HpHRP) for peptide fractionation. Remaining dried-pool aliquots were stored at –80 ^o^C for repeated analysis if required.

### HpHRP fractionation

HpHRP was performed using an Agilent high performance liquid chromatography (HPLC) 1200 system (Agilent, Santa Clara, California). Reversed-phase chromatography buffers (buffer A; 0.1% (v/v) ammonium hydroxide in HPLC grade water and buffer B; 0.1% (v/v) ammonium hydroxide in acetonitrile) were made fresh. Each iTRAQ-labelled pool sample was re-suspended in 900 μL of 3% (v/v) buffer B and loaded onto a HpHRP column (ZORBAX 300Extend-C18 4.6 × 150 mm 3.5 μm, Agilent) for 40-min with a flow of 1 mL/min at 3% (v/v) buffer B. The peptides were then eluted using the gradient as follows (minutes:%B); 0:3, 5:3, 30:27, 35:50, 36:100, 41:100, 42:3. A total of 86 fractions were collected in a 96-well plate, which was dried in a centrifugal evaporator (Eppendorf) and stored at –20 °C prior to LC-MS/MS analysis.

### Low-pH LC-mass spectrometry data acquisition

Each fraction was re-suspended in 27 μL of 97% water + 3% acetonitrile + 0.1% trifluoroacetic acid (TFA; v/v/v) and 9 μL was injected into a nano-Acquity UPLC system (Waters). Peptides were trapped on a nanoACQUITY 2G-V/M Trap Sym C18 5 µm 180 µm × 20 mm (Waters) and washed at a flow rate of 7.5 μL/min for 10 min. Peptides were then eluted and chromatographed using a nanoACQUITY BEH300 C18 1.7 μm 75 μm × 250 mm (Waters) at 300 nL/min using following gradient profile (minutes:%B); 0:3, 3:3, 91:40, 93:90, 108:90, 109:3, 130:3. The buffers used were: buffer A: 97% water + 3% acetonitrile + 0.1% formic acid and buffer B: 100% acetonitrile + 0.1% formic acid (v/v).

The eluent was directed into an ESI microionspray II source of a QSTAR Elite Q-TOF spectrometer (AB SCIEX) scanning in MS from 400 to 1200 m/z. Multiply charged peptides (2 + to 4 + ) were selected for MS/MS analysis (110–1200 m/z). The information-dependent acquisition (IDA) settings were: four precursors per cycle and cycle times (MS 0.75 s, MS/MS1 0.75 s, MS/MS2 0.75 s, MS/MS3 1 s and MS/MS4 1 s). Selected peptides were fragmented twice and then dynamically excluded for 90 s. The resulting data were searched against the human component of the Swissprot database (release 2013_03) using Protein-Pilot v4.0 (AB SCIEX). Search parameters were: iTRAQ 8-plex, trypsin; cys alkylation, iodoacetamide; search effort, thorough. A total of 40,466 proteins were searched. To perform FDR analysis on the protein identification, the search database was reversed and concatenated with the forward database and used as the search DB within ProteinPilot. FDR was determined by calculating the number of reverse ‘hits’ as a proportion of ‘forward’ hits using the dedicated worksheet exported from the search software.

### Data processing

Bayesian protein-level differential quantification was performed separately for each brain region using v1.0.0 of the in-house developed software BayesProt (https://github.com/biospi/bayesprot/releases/tag/v1.0.0). An earlier version of this technique was presented in Freeman et al.^66^, which combined Protein-Pilot (AB SCIEX) sample normalisation (‘bias correction’) with a Bayesian linear mixed-effects model implemented with the MCMCglmm R Package^67^. Analysis of each brain region in isolation adds strength to our comparison of protein expression changes across multiple regions, as these were identified and quantified independently.

Since iTRAQ measurements from Time-of-Flight instruments are recorded as discrete ion counts, and technical/biological variation are assumed log-normal, we adopted a generalised linear mixed model (GLMM) with Poisson likelihood and log-link, where each protein was modelled separately using peptide measurements unique to that protein. The sample normalisation factors represent the mass spectrometer’s exposure to each sample, and hence were included as a fixed offset within the model. The current version of BayesProt additionally (i) enables estimation of both biological and digestion variance through the incorporation of multiple digests for a single sample (i.e., the six reference pool digests), (ii) negates the need for Protein-Pilot normalisation by implementing a two-stage GLMM and (iii) provides a simplified Markov Chain Monte Carlo (MCMC) mixing criterion for both stages.

In both stages: (a) for each peptide a separate random digest effect is fitted, which has the effect of weighting each peptide’s contribution to the protein-level quantification by its reproducibility across digests; (b) the set of measurement channels within each iTRAQ spectrum are each assigned (i) a baseline fixed effect to account for varying selection/ionisation/fragmentation efficiencies across spectra, and (ii) an independent log-normal residual variance to account for over-dispersion due to background contamination and incorrectly identified spectra. In stage one, we also model the interaction between LC-MS/MS run and iTRAQ channel as a fixed effect, i.e., within each run, we infer the protein-level log ratio between iTRAQ channel 113 and channels 114, 115, 116, 117, 118, 119 and 121. For each channel relative to 113, the result is a set of posterior probability distributions, one for each protein in the study; these are combined to derive a posterior distribution for the median log ratio for each channel relative to 113, which is taken as the inferred sample normalisation factors. To construct the PCA plots presented in Supplementary Figure 1, the protein-level log ratios for all proteins with measurements across all three 8-plexes were first normalised using these sample normalisation factors. Subsequently, for each protein ‘variable’, the resulting sample ‘observations’ were then centred and scaled by the mean standard deviation of their posterior distributions, before final input into the R ‘prcomp’ function to generate the principal components.

In stage two, rather than using point estimates of the normalisation factors as fixed sample offsets, a set of sample fixed effects are fitted, which have prior distributions set to the means and variances of the inferred median log ratio distributions. In addition, in stage two we specify the full experimental design: (a) protein-level differential expression fold change between cases and controls is fitted as a condition fixed effect (with control as baseline); (b) due to unequal biological variance across cases and controls, subject is treated as two random effects, one for control samples and one for cases. Using the inferred posterior distribution of the condition fixed effect, we performed a one-sided significance test on the posterior probability that the mean fold change is either above or below ± 1.05—i.e., at least a 5% change from control—denoted as *P* (1.05 fc). The reciprocal of this posterior probability represents the local FDR (lFDR), the probability that this specific test is a false discovery. In this study, we defined significance using a global FDR threshold of 5%, i.e., the largest set of proteins with an average lFDR ≤ 5% were deemed significant and hence delivered to downstream pathway analysis. The condition fixed effect posterior distributions, FDRs and descriptive statistics (mean log ratio plus 95% highest posterior density interval) for every protein across all regions are presented online (www.manchester.ac.uk/dementia-proteomes-project). Posterior distributions of per-sample protein quantifications are also presented, derived from the latent variables of the sample random effects.

Residual variances were assigned inverse-Gamma priors, whereas random effects were assigned parameter-expanded Cauchy priors. The model was tested with different prior scale factors to establish that the priors were not informative to the outcome. In stages one and two, the model was run with 10 and 100 MCMC chains per protein, respectively, each chain consisting of 10,000 samples preceded by 3000 burn-in samples. Mixing was assessed using Warnes & Raftery’s MCGibbsit run-length diagnostic, combining the estimate error-bounding approach of Raftery and Lewis with the between chain variance verses within chain variance approach of Gelman and Rubin (https://cran.r-project.org/web/packages/mcgibbsit/index.html).

For a protein to be considered quantified sufficiently well to be included in downstream pathway, correlation and comparative analyses, we require identification and quantification from at least three spectra. This quality control is important when making comparisons across datasets as it ensures that only high-quality protein quantitation is taken forward into comparative studies, reducing ‘noise’.

### Data analysis

Processed protein-level data were analysed through a range of software tools. Data alignment, filtering and characterisation was initially performed in Microsoft Excel. Heat maps were constructed using Cluster 3.0 (http://bonsai.hgc.jp/~mdehoon/software/cluster/software.htm) and viewed using Java TreeView (https://sourceforge.net/projects/jtreeview/files/;^68^). Venn diagrams were built using the Interactive Venn tool (www.interactivenn.net;^69^). The Isomap algorithm^70,71^ was implemented in Qlucore Omics Explorer (version 3.2, Qlucore, Lund, Sweden).

### Network analysis

Pathway enrichment analysis was performed for each brain region independently using Ingenuity Pathway Analysis (Qiagen), selecting the user dataset as the background and considering only relationships, which had been experimentally observed or predicted with ‘high’ confidence, and was limited to human interactions. Following analysis, significant pathways were reviewed and those which contained genes, which formed a complete subset of another pathway were removed. In parallel, we performed functional annotation clustering analysis for each brain region using online DAVID (https://david.ncifcrf.gov/;^72,73^) with custom classification stringency setting; similarity term overlap = 5, similarity threshold = 0.95, initial group membership = 3, final group membership = 3, multiple linkage threshold = 0.5, EASE = 1.0 and Benjamini correction. Enrichment score ≥ 1.3 was considered significant and highlighted in supplementary data 5. Protein–protein interaction networks were analysed for proteins that were uniquely altered in CB only, using online STRING (https://string-db.org;^74^) at default setting.

To identify key regulators of protein expression, a correlation matrix of protein expression across the six brain tissue samples was generated in Qlucore Omics Explorer and modified in R to only contain proteins with a│0.9│*r*-value. The network was visualised in Cytoscape^75^. Protein modules with correlated expression were identified using the Moduland algorithm^50^ and arranged in a hierarchy based on their network centrality.

## Supplementary Information

Supplementary Information

Supplementary Data 1

Supplementary Data 2

Supplementary Data 3

Supplementary Data 4

Supplementary Data 5

Supplementary Data 6

Description of Additional Supplementary Files

## Data Availability

All raw mass spectral data, along with extracted.mgf peaklists and ProteinPilot.group results files generated during this study are available via the PRIDE data repository, with each brain region submitted independently to reflect the way in which the study was performed. PRIDE accessions are: Hippocampus—PXD008739; Entorhinal cortex—PXD008806; Cingulate gyrus—PXD008779; Motor cortex—PXD008807; Sensory cortex—PXD008753; Cerebellum—PXD008755. We recognise that these data require specialist interpretation. To support data sharing, we have also made available the outputs of our initial MS analysis (after ProteinPilot database searching for peptide/protein identification and peptide relative quantification, but before Bayesian inference) by depositing the Protein Summary (protein identification data) and Peptide Summary (peptide identification data), along with the raw MS peak lists, with the Open Science Framework (https://osf.io), which can be accessed by the following 10.17605/OSF.IO/6BXJQ. All fully processed data are available via the Supplementary Data associated with this Article, and online in a searchable format, along with probability distribution plots for each protein, at www.manchester.ac.uk/dementia-proteomes-project.
