# Supplementary material for: Regional protein expression in human Alzheimer’s brain correlates with disease severity
Source: Commun Biol. 2019 Feb 4;2:43. doi: 10.1038/s42003-018-0254-9 (PMC6361956; doi:10.1038/s42003-018-0254-9)

Supplementary Figure 1a

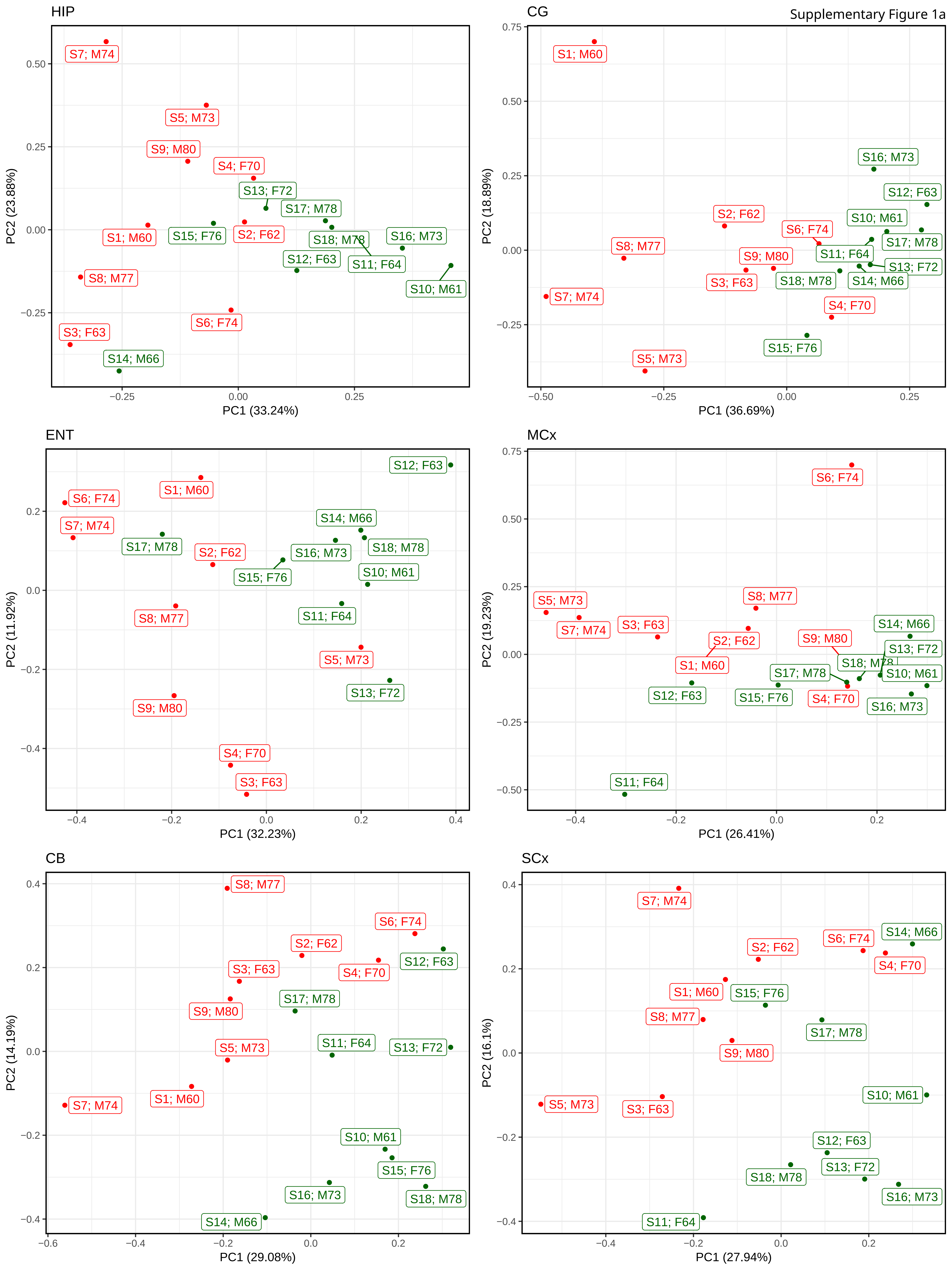

Supplementary Figure 1b

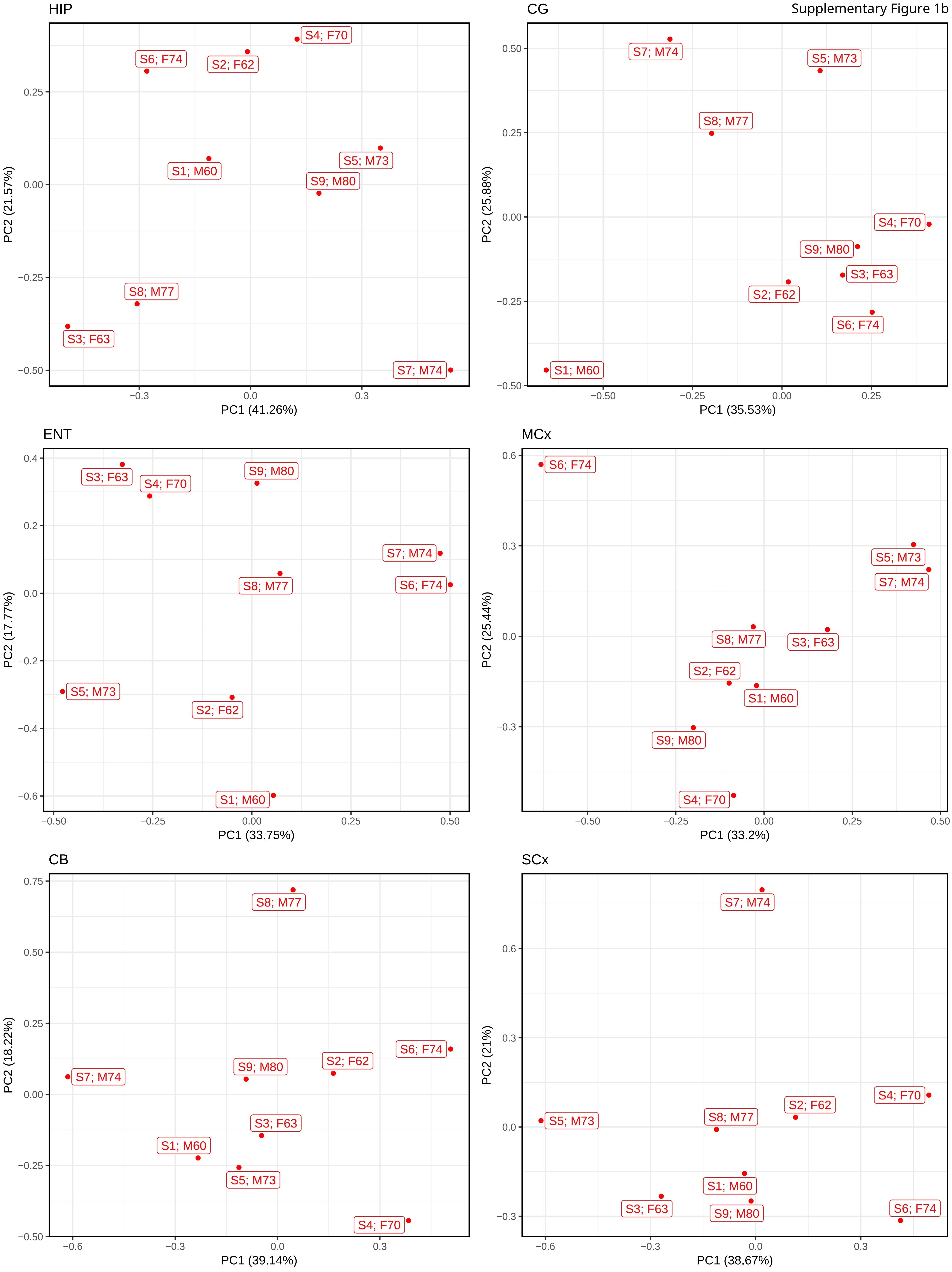

Supplementary Figure 1. Principal Component Analysis plots of sample 'observations' against protein-level quantifications (log ratios with first reference sample in each 8-plex as the denominator – see Methods), illustrating clustering by condition in each of seven brain regions, and no clear confounding clustering by age or gender. Figure 1a shows this clustering for all samples in the study. Figure 1b shows a secondary analysis on AD samples only, to check for gender-related differences in the disease cohort.

Supplementary Information : User guide for navigating online datasets.

To aid in the dissemination and utility of these data, we have established an online repository for this study from which it is possible to search our datasets for specific genes/proteins of interest and determine both their relative quantity, and the quality of that data, in our study. This repository is housed at www.manchester.ac.uk/dementia-proteomes-project.

Here we provide a brief overview of this resource, and how it can be used and how data are interpreted.

Data is searched via a free text search which will search either the gene name, or any word in the protein description, SWISS-Prot ID or accession number.

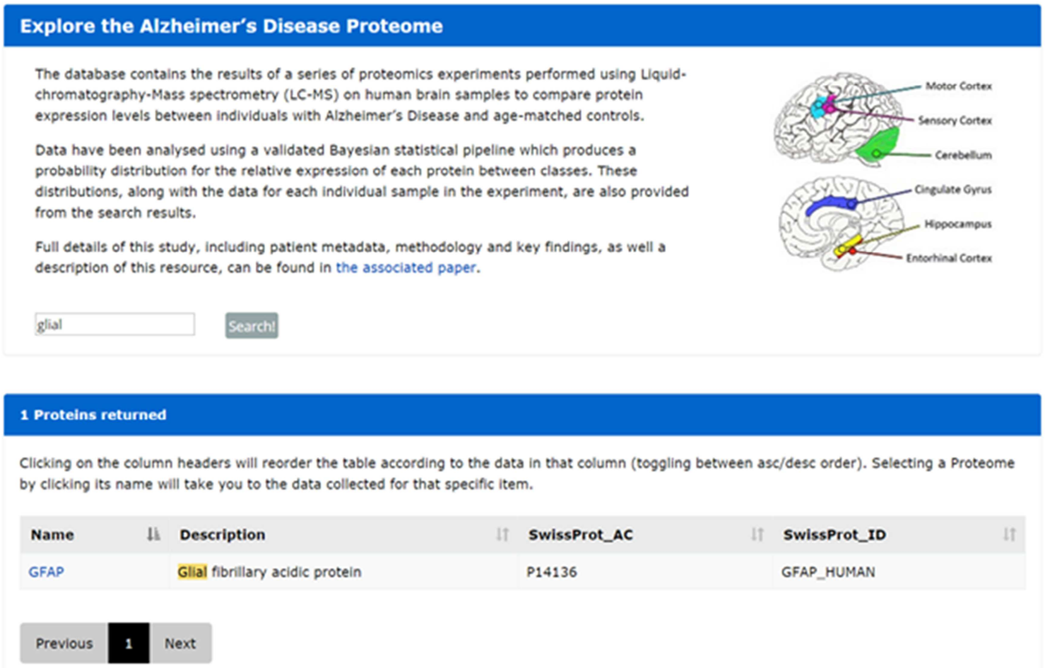

Selecting the Name of the protein of interest opens a table (below) showing the regions for which we have quantification data for that protein, and associated data as follows:

GFAP

AC : **P14136**
ID : **GFAP_HUMAN**
Description : **Glial fibrillary acidic protein**

Brain Area	Peptides	Spectra	Log_2(fc)	Lower	Upper	LocalFDR	GlobalFDR
Hippocampus (HIP)	95	1829	0.529149485	-0.066167874	1.103791236	0.0573	0.021314341
Motor Cortex (MC)	44	590	0.198755814	-0.247789602	0.661911914	0.2655	0.140215328
Sensory Cortex (SC)	57	821	0.095872844	-0.330819011	0.544217367	0.448	0.259713819
Cerebellum (CER)	61	794	0.211085361	-0.153213647	0.570785583	0.1952	0.087537702
Entorhinal Cortex (ERC)	70	1045	0.49231705	-0.078521237	1.084060479	0.0681	0.028219935
Middle Temporal Gyrus (MTG)	49	901	0.524556034	0.096108562	0.951143024	0.0196	0.007880435
Cingulate Gyrus (CG)	54	1211	0.46881308	-0.168811081	1.094320968	0.0961	0.040010067

Peptides – the number of distinct peptides identified associated with the protein of interest.

Spectra – the number of distinct MS/MS spectra associated with the protein of interest.

Log_2(fc) – The Log to base 2 of the fold change (fc) difference between Alzheimer's Disease and Control tissues. Positive numbers imply a relative increase in AD, negative numbers imply a relative decrease.

Lower and Upper – Describe the lower and upper 95% credible intervals for the protein relative quantitation in that region.

LocalFDR and GlobalFDR – One minus the probability that this protein exhibits at least a 5% deviation from control levels in AD (local FDR) and a calculated global FDR demonstrating the average of localFDRs across the dataset.

To explore these data in more depth, the user can select the Log2(fc) ratio for each condition and view the probability density plot for that specific protein in that region. Note that the algorithm calculates a localFDR (1-p(protein differs from control by at least 5%) both for upregulation and downregulation. However for each protein only the lowest local FDR i.e. most likely result is listed.

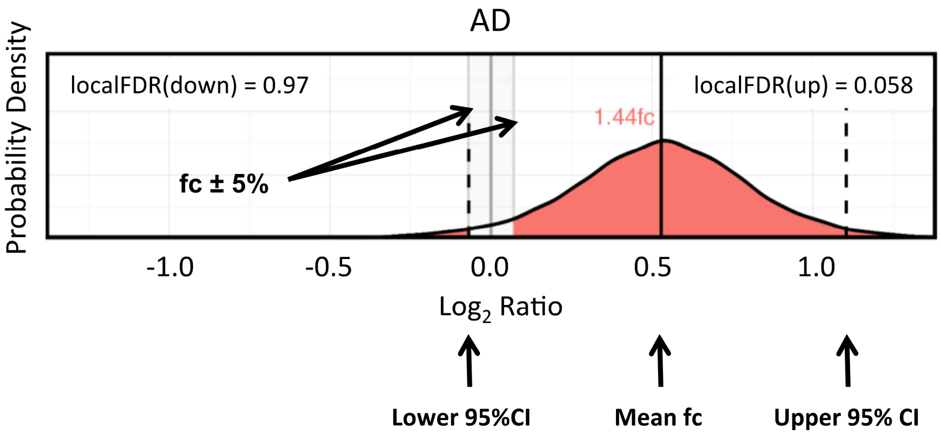

To view variability across all samples in the study, the user can also select the region name. This allows them to view the posterior distribution of each protein on a per-sample basis.

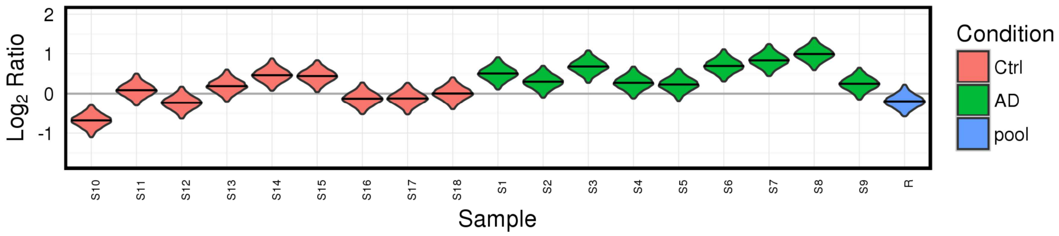

Supplement: Supplementary file 1 — Supplementary Information [file 42003_2018_254_MOESM1_ESM.pdf]
