# Supplementary material for: Regional protein expression in human Alzheimer’s brain correlates with disease severity
Source: Commun Biol. 2019 Feb 4;2:43. doi: 10.1038/s42003-018-0254-9 (PMC6361956; doi:10.1038/s42003-018-0254-9)
Supplement: Supplementary file 8 — Description of Additional Supplementary Files [file 42003_2018_254_MOESM8_ESM.docx]

**Description of Additional Supplementary Files**

**File Name**: Supplementary Data 1

**Description**: Quantitative data on all proteins identified with an FDR below 1% for each brain region. Columns A-E contain protein identification information. Columns G-R contain the Protein ID for each protein in each regions from our Bayesian analysis (a ranking given by the Bayesian analysis ordering the proteins by the number of quantified spectra), and the N number for each protein from the raw ProteinPilot analysis for each of the six regions (an alternate ranking with 1 having the highest identification score, i.e. most unique high confidence peptides). Columns T-Y list the number of distinct peptides associated with each protein in each region, and columns AA-AF contains the number of distinct MS/MS spectra associated with each protein for the purposes of quantification. The remaining columns contain the quantitative data for each region. Briefly, for each region these data are: ‘mean’ - log2(AD:Control); ‘lower’ and ‘upper’ – upper and lower 95% credible intervals; ‘localFDR’ and ‘globalFDR’ – local and global Bayesian false discovery rates. More detail on these values is provided in Supplementary Information and the description of our online database.

**File Name**: Supplementary Data 2

**Description**: Data used for the construction of the Edwards-Venn diagram in Figure 2. All genes identified in all six regions are listed in column A. Columns D-I show our call in terms of differential expression between AD and control. Columns K-P show a filtered list of genes whose expression changes in each region. Columns S-U list each changing gene according to the intersection in the Edwards-Venn diagram to which they were assigned.

**File Name**: Supplementary Data 3

**Description**: Major protein changes between AD and control brain. 128 proteins were shown to be significantly differentially expressed between AD and control in at least 5/6 regions studied. The gene names are listed, along with the direction of change. All proteins change in a consistent direction in all regions. Column C provides a brief description of the role of each protein. Column D describes whether we have been able to find a reliable link between that protein and AD in the literature, though a combination of literature searching using PubMed and Google – the latter strategy includes in the results genes names which are provided in tables (in the main article, not in supplementary tables), but are not mentioned by name in the article text. A doi for a representative reference is provided in Column E.

**File Name**: Supplementary Data 4

**Description**: Output from pathway-enrichment analysis. For each brain region detailed data regarding the pathway enrichment analysis is presented. For each region, significant pathways (P<0.05) are presented and are ordered into related groups. For each pathway the p-value is presented along with the percentage of protein in that pathway which are shown to be differentially expressed and a IPA-generated z-score which is designed to calculate the relative activation (or inhibition) of each pathway, where possible. All genes associated with that pathway are listed and colour-coded according to whether they are upregulated in AD (red) or down-regulated (green). The total numbers of up and down-regulated proteins in each pathway are also provided.

**File Name**: Supplementary Data 5

**Description**: Comparison with data from Seyfried et al. A) Ingenuity pathway analysis of data from Seyfried et al14 from proteins shown to be differentially expressed in AD versus control in dorsolateral prefrontal cortex (p<0.01). For each pathway the p-value is presented along with the percentage of protein in that pathway which are shown to be differentially expressed and a IPAgenerated z-score which is designed to calculate the relative activation (or inhibition) of each pathway, where possible. B) Direct comparison of all 256 protein changes observed in AD versus control (p<0.01) by Seyfried at al (columns F, G) with corresponding quantitation data from our study as described in Supplementary Table 2.

**File Name**: Supplementary Data 6

**Description**: Results from DAVID analysis of pathways enriched specifically in Cerebellum.
